# Location of mechanically-evoked referred sensations within the trigeminal region are not altered following a heterotopic painful stimulus

**DOI:** 10.1038/s41598-022-24510-0

**Published:** 2022-12-07

**Authors:** Fernando G. Exposto, Miranda Huang, Talita Haasnoot, Michail Koutris, Frank Lobbezoo, Karina H. Bendixen, Peter Svensson

**Affiliations:** 1 Section of Orofacial Pain and Jaw Function, Department of Dentistry and Oral Health, Aarhus University, Vennelyst Boulevard 9, 8000 Aarhus C, Denmark; 2 Scandinavian Center for Orofacial Neurosciences (SCON), Aarhus, Denmark; 3 Department of Oral Kinesiology, Academic Centre for Dentistry Amsterdam (ACTA), University of Amsterdam and Vrije Universiteit Amsterdam, Amsterdam, The Netherlands; 4 Faculty of Odontology, Malmø University, Malmö, Sweden

**Keywords:** Pain, Mandibular muscles

## Abstract

To investigate whether the location, area and frequency of referred sensations occurring during palpation of the masseter muscle can be influenced by application of a conditioning painful stimulus to the temporalis muscle. Thirty healthy participants were included in this cross-over study, performed in two sessions with > 48 h in between. At each session, palpation of the masseter muscle was performed before and after 0.2 ml of glutamate (1 mol/L) or isotonic saline (control) were injected into the anterior portion of the temporalis muscle. Palpation of the masseter muscle was done using four different forces (0.5 kg, 1 kg, 2 kg and 4 kg). Participants rated the perceived intensity of the palpation and any referred sensations on a 0–50–100 numeric rating scale, the perceived pain intensity following the injections on an electronic visual analogue scale and drew any referred sensations they experienced. No difference in referred sensations location, area and frequency was shown r during palpation either before or after injections (*P* > 0.05). A moderate correlation was found between perceived sensation scores and referred sensations intensity for the temporalis muscle following glutamate injection (*r* = 0.407, *P* < 0.05). Moreover, significantly more participants reported referred sensations for glutamate injections into the temporalis muscle when compared to isotonic saline (*P* < 0.05). Finally, a significant decrease in the perceived intensity of palpation of the masseter muscle was seen after glutamate injection in the temporalis muscle (*P* < 0.05). In the current study, location, area and frequency of referred sensations following mechanical stimulation of the masseter muscle were not altered by the application of a painful stimulus to the temporalis muscle. In addition, there seems to be a positive relationship between painful stimuli and referred sensations frequency and intensity elicited from the temporalis muscle.

## Introduction

Referred sensation has been known and described in the literature for many years and is defined as “a sensation or pain felt at a site remote from the site of origin/stimulation”^1^. It is frequently present in several musculoskeletal pain conditions^2^ and commonly occurs within the orofacial region^3^. For example, pain from muscles may refer to other locations like the teeth^4,5^, which can confuse clinicians and might even lead to misguided diagnoses and incorrect treatment in case no local cause of the sensations or pain can be found, leaving the actual cause untreated^6^.

To this date, the etiology and pathophysiology of referred sensation are still not fully elucidated. Most researchers agree that both peripheral and central mechanisms may mediate referred sensations^1,2,7,8^. In both the trigeminal and spinal systems studies have shown that painful stimuli, for example by injection of algogenic substances such as hypertonic saline or glutamate^9–12^, or by application of pressure to the muscle^9^, can provoke referred sensations. It has also been shown that a positive relation exists between the intensity of nociceptive input and the frequency of eliciting referred sensations^9,13^. Within the trigeminal system, most studies have assessed referred sensations from the masseter muscle and as such, it is unclear whether the same findings occur for the temporalis muscle.

Experimental studies have demonstrated that pain may refer to areas with previous pain experience, for example after a tooth extraction,^14^ persistent pain, such as the temple in headache patients^15^ or even in patients who have recovered from a fracture^16,17^. Despite this, it has not been assessed whether an experimental heterotopic noxious conditioning stimulus, such as glutamate in the temporalis muscle can alter the location or frequency of referred sensations when pressure to the masseter muscle is applied.

Therefore, the primary aim of this study was to assess if the location, area, and frequency of referred sensations originating in the masseter muscle as well as the mechanical sensitivity of the masseter muscle would be influenced by the application of a painful conditioning stimulus to the temporalis muscle. The hypotheses were that after application of a painful stimulus to the temporalis muscle the area and frequency of referred sensations originating from the masseter muscle would increase when compared to before the stimulus to the temporalis muscle. In addition, that referred sensations would refer more frequently to the temple region, the area where the painful conditioning stimulus was applied, after the painful stimulus than before. Finally, that there would be a decrease in mechanical sensitivity of the masseter muscle following a painful stimulus to the temporalis muscle. The secondary aim was to assess if a relation exists between the painfulness of a stimuli applied to the temporalis muscle and referred sensations frequency and intensity. The hypothesis being that the more painful a stimulus is the higher the chances of eliciting referred sensations.

## Material and methods

### Participants

A total of 34 healthy volunteers participated in this study. A sample size estimate suggested that with 20 participants we would be able to detect a 25% difference in frequency of referred sensations (primary outcome) before and after glutamate injection into the temporalis muscle with α = 0.05, and β = 0.2. Despite this and due to some participants possibly not experiencing referred sensations we opted to increase the number of participants to 30. Participants were recruited through advertisement flyers that were hanged at the Aarhus University Campus and via a social media website (Facebook). All participants were screened by two examiners (MH and TH).

The participants had to be > 18 years of age and show no signs of symptoms of temporomandibular disorders (TMD) according to the Diagnostic Criteria for Temporomandibular Disorders (DC/TMD) (with the exception of non-painful temporomandibular joint sounds)^18^. Pregnant women and participants with orofacial inflammatory diseases, orofacial pain or fibromyalgia were not included in this study. Use of any regular medication and/or recreational drugs as well as pain medication 24 h prior to the start of the experimental study were also exclusion criteria.

Participants were provided with information on the study, and they had to give their written informed consent before they were included in the study. After the experiment, all participants received a monetary compensation for their time and effort. The experiment was performed at the Department of Dentistry and Oral Health, Aarhus University, Denmark. The study was approved by the Central Denmark Region Research Ethics Committee (Ref. Nr. 1-10-72-246-17) and was performed in accordance with the Helsinki Declaration.

### Experimental protocol

The study was performed as a randomized, double-blind, and placebo-controlled trial. It was performed in 2 sessions with at least 48 h in between.

**Figure 1:**
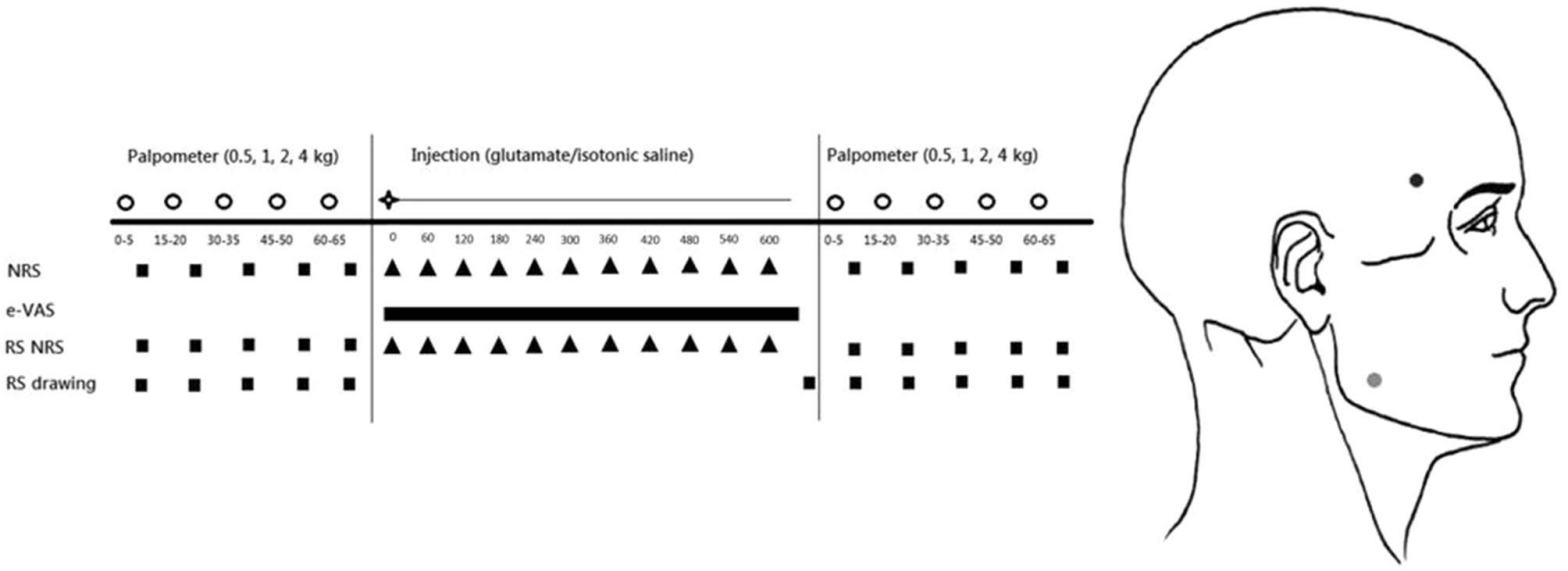
A schematic illustration of the experimental protocol. The white circles indicate one of the repetitions of the forces. The injection (star) was either glutamate or isotonic saline. The numbers indicate the time in seconds. The black squares indicate when the different parameters were assessed. The black stripe indicates a continuously-updated e-vas scale. The referred sensation intensity (RS NRS) and referred sensation drawing (RS drawing) were only collected when a referred sensation was present. Both sessions were performed with at least 48 h between them. The place of injection (black dot) and palpation (grey dot) can be on the left or right side of the face. Copyright International RDC/TMD Consortium Network. Available at http://www.rdc-tmdinterna/onal.org. Version 12May2013. No permission required to reproduce, translate, display, or distribute.

Each session was divided into three parts. The experimental protocol was performed on the muscles on one side of the face (left or right) which was randomly chosen. Each appointment consisted of a baseline mechanical sensitivity assessment of the masseter muscle, followed by an injection of either glutamate or isotonic saline into the temporalis muscle, and ended with a post-injection mechanical sensitivity assessment of the masseter muscle. During the second session, the experiment was repeated in the same way changing only the injected substance. The protocol and sequence procedure are explained in Fig. 1.

### Mechanical sensitivity assessment

The mechanical sensitivity of the masseter muscle was assessed at the start of each session and approximately 15 min after the glutamate/isotonic saline injection into the anterior portion of the temporalis muscle^10^ (Fig. 1). For all participants and in all sessions the bulkiest part of the masseter muscle was identified by asking the participants to clench their teeth. This portion of the masseter muscle was then palpated with a standardized and previously validated instrument, the palpometer (Palpeter®, Sunstar Suisse SA) which has a probe with a contact area of 1cm^2^^19,20^. Four different forces (0.5 kg, 1.0 kg, 2.0 kg and 4.0 kg) were used to palpate the masseter muscle in a randomized order, and each force was repeated a total of five times consecutively. Each palpation lasted 5 s^18^, followed by a 10 s interval where the participants were asked to rate their perceived sensation intensity on a 0–50-100 numeric rating scale (NRS) (where 0 = no sensation, 1–49 = any sensation that was not pain, 50 = pain threshold, 50–99 = painful, 100 = worst pain imaginable)^21–24^ and to report if they perceived any referred sensations. If a referred sensation was present, participants were asked to rate the referred sensation intensity on the same 0–50–100 NRS and draw its location on an anatomical map of the head. Sensations were considered referred if the participants reported a sensation that was felt in different areas of the head, face, mouth and/or neck other than the site being palpated^25^. If participants reported a RS they were instructed to point to all areas in which they felt the sensation and the examiner determined whether the sensation was felt in the same structure as the palpation, in this case the masseter muscle, or a different structure.

During the assessments, the participants were instructed to keep their mandible at rest and the hand of the examiner was placed on the contralateral side to prevent the mandible from moving. To make sure the participants understood the concept of the 0–50–100 scale, it was explained to them thoroughly and as many times as needed until the participants felt they understood the scale. In addition, a practice run was done in the thenar region to confirm the participants understood the scale.

### Chemical stimulation

A 27-G needle was used to inject 0.2 ml of glutamate solution (1 mol/L) or a buffered isotonic saline solution in 10 s into the temporalis muscle. This was given as a bolus following a previously described protocol^10,12^ and was injected into the bulkiest part of the anterior portion of the temporalis muscle and was identified during tooth clenching.

Immediately after the injection, the participants were asked to continuously rate their pain intensity in the temporalis muscle on a 0–10 cm electronic visual analog scale (eVAS) where 0 represented no pain and 10 represented the worst pain imaginable for 10 min following the injections. Additionally, each minute for 10 min, they were also asked to rate their perceived sensation intensity in the temporalis muscle on the same 0–50–100 NRS, whether they were experiencing any referred sensation and if so to rate the sensation on the 0–50–100 NRS. After the 10 min, they were asked to draw the location of any referred sensation they had experienced on an anatomical map of the head. Sensations were considered referred if the participants reported a sensation that was felt in different areas of the head, face, mouth and/or neck other than the site of the injection^25^. If participants reported a RS they were instructed to point to all areas in which they felt the sensation and the examiner determined whether the sensation was felt in the same structure as the injection site, in this case the temporalis muscle, or a different structure.

### Referred sensation location and area

The anatomical map, included in the DC/TMD^18^, was used to assess the location and area of referred sensations. It contained extraoral projections of the face and neck (lateral, frontal and occipital), and an intraoral projection in order to include each facial area. Referred sensation or pain was defined to the participant as a sensation or pain felt outside the anatomical structure being palpated or injected^18^. Therefore, spreading pain was not included in the analysis. For both mechanical and chemical stimulation, if participants reported referred sensations they were asked to show the examiner where they had experienced the referred sensation who then judged whether this was outside of the boundary of the assessed muscle. The outcome of interest for the referred sensations location was whether pain or sensations were referred to the temple region after an injection to the temple region more frequently than before injection. For the referred sensations area the outcome of interest was whether average referred sensations areas were bigger after an injection to the temple than before.

### Randomization

In the first session, half the participants received a glutamate injection and the other half received an isotonic saline injection. A balanced randomization procedure was carried out for the injection side (left or right), the order of injections (glutamate or isotonic saline first), the order in which the different forces were applied, and the examiner (TH or MH) that was going to perform the experimental part of the study. This was done using an online randomization tool (randomization.com).

Outcome measures.

The primary outcome measures of this study were as follows:

1. Differences in RS location, area and frequency provoked by palpation of the masseter muscle compared before and after a painful (glutamate) or control (isotonic saline) injection into the temporalis muscle.
2. Difference in perceived sensation intensity following palpation of the masseter muscle compared before and after a painful (glutamate) or control (isotonic saline) injection into the temporalis muscle.

The secondary outcome of the study was as follows:

1. To assess the relationship between the painfulness of a stimuli applied to the temporalis muscle and referred sensations frequency and intensity originating in the temporalis muscle.

### Statistics

Q–Q plots were used to assess the normal distribution of the data, which were all found to be normally distributed. For the primary outcomes, McNemar’s test was used to compare differences in frequency of referred sensations and referred sensation location before and after injections. In addition, a 2-way repeated measured analysis of variance (ANOVA) was used to compare differences in area of referred sensations: substance (glutamate and isotonic saline) and session (baseline and post-injection). Finally, a 3-way repeated measures ANOVA was performed to compare the perceived sensation intensity of the masseter muscle before and after injections with the main factors: substance (glutamate and isotonic saline), session (baseline and post-injection) and force (0.5 kg, 1.0 kg, 2.0 kg and 4.0 kg). Tukey HSD tests were used for post-hoc comparisons of the ANOVAs.

For the secondary outcomes, a two-way repeated measures ANOVA was performed to compare the chemical perceived sensation intensity scores measured during the 10 min after the injections with the main factors: substance (glutamate and isotonic saline) and time (1 through 10 min). McNemar’s test was used to test for differences in referred sensations between glutamate (painful stimulus) and isotonic saline (non-painful stimulus) for the temporalis muscle. Finally, a Pearson correlation was performed to assess the relation between the perceived sensation intensity scores and the intensity of referred sensations after injection to the temporalis muscle. *P* values smaller than 0.05 were considered significant.

## Results

### Participants

Data from 34 participants were collected, but only the data of 30 participants were analyzed. The included group of participants consisted of 17 men and 13 women. The average age was 26.0  ± 7.9 years (SD). Four participants were excluded from the study because two did not feel pain during the glutamate injection, and two withdrew from the study following the first session due to experiencing too much pain following injection.

### Referred sensations following mechanical stimulation of the masseter muscle

**Figure 2:**
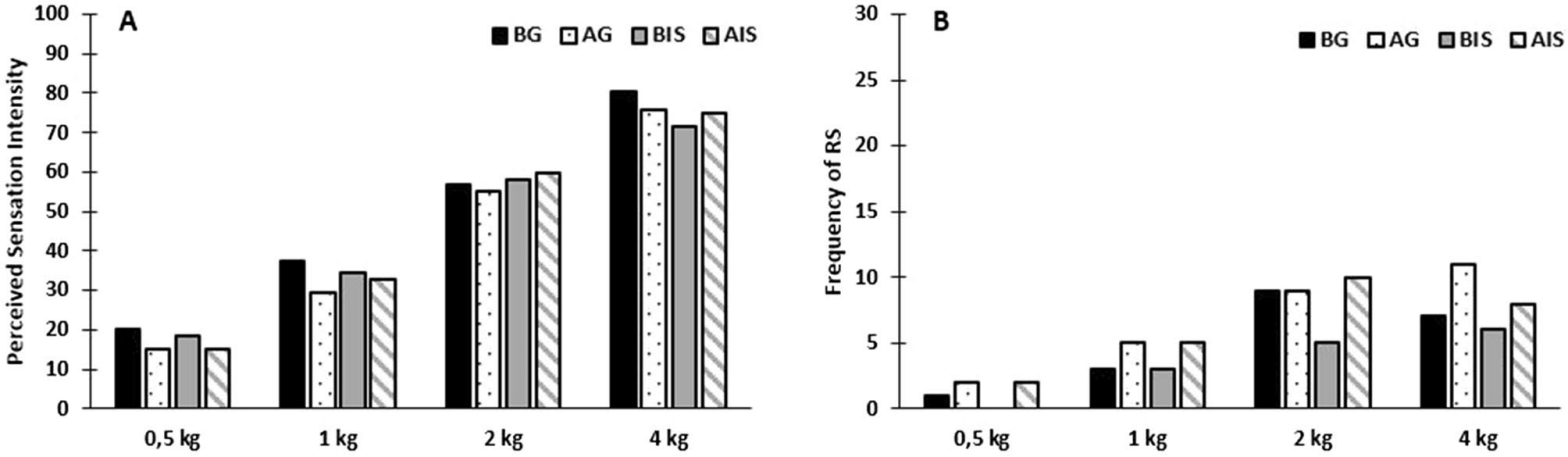
Referred sensations and perceived sensation intensity following mechanical stimulation of the masseter muscle. No significant difference was found between before and after injections regarding perceived sensation intensity (**A**) and referred sensations (**B**) following palpation of the masseter muscle. BG—Before glutamate; AG—After glutamate; BIS—Before isotonic saline; AIS—After isotonic saline.

During the mechanical stimulation, 21/30 participants (70%) experienced referred sensations. The prevalence of referred sensation was 4/30 (13%), 8/30 (27%), 16/30, (53%) and 16/30 (53%) for 0.5 kg, 1 kg, 2 kg and 4 kg, respectively (Fig. 2).

There was no difference in the frequency of referred sensations during mechanical stimulation between the baseline and post-injection sessions for either substance or any of the forces (McNemar, *P* > 0.05) (Fig. 2).

No significant difference was seen for the area of referred sensations following mechanical stimulation between before and after injections for either glutamate or isotonic saline (2-way ANOVA, substance × session, *F* (1, 29) = 1.19, *P* = 0.28)*.*

No significant difference was seen for referred sensations location following mechanical stimualtion before and after both the glutamate or isotonic saline injections (McNemar, *P* > 0.05) meaning that referred sensations following mechanical stimulation did not refer more frequently to the temple after injections.

### Referred sensations following injections into the temporalis muscle

**Figure 3:**
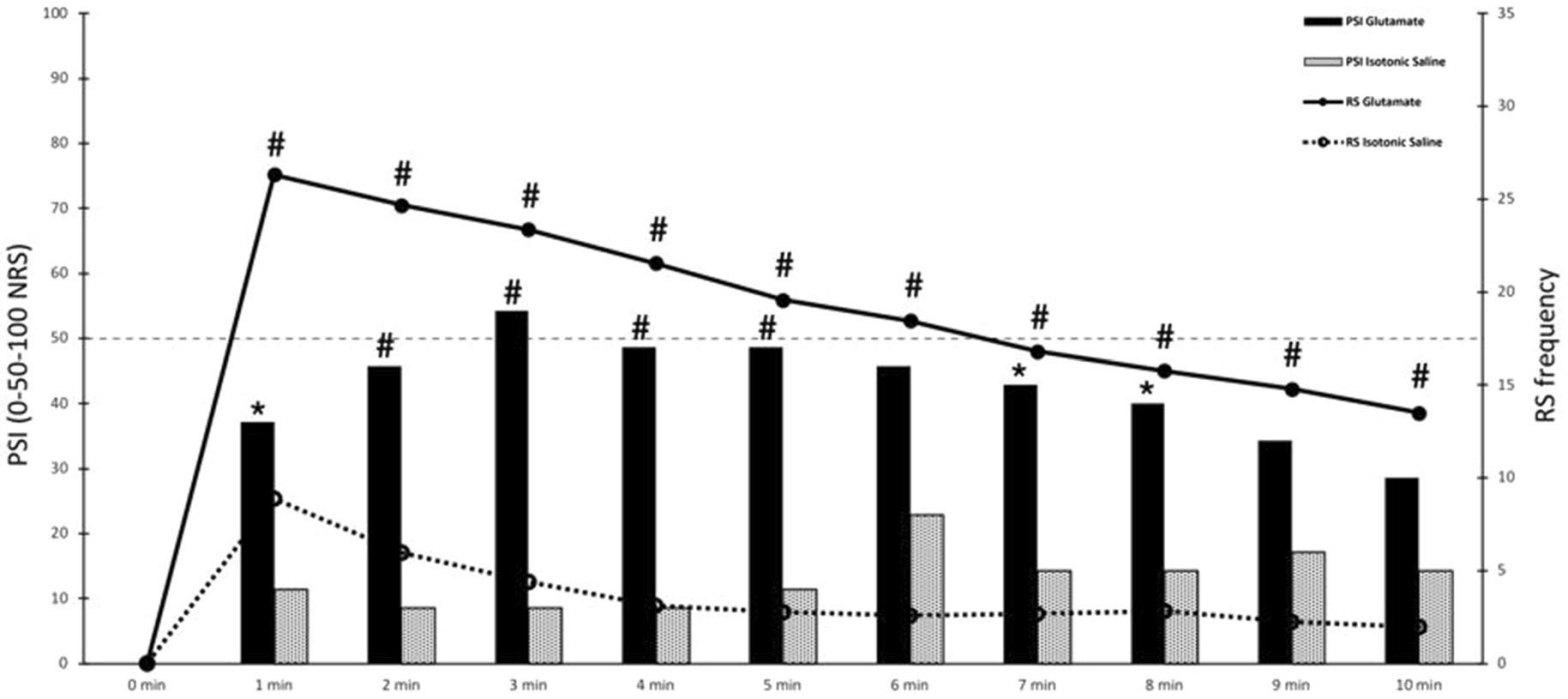
Referred sensations and perceived sensation intensity following injections into the temporalis muscle. A visual representation (graph) of the recorded referred sensation (RS) frequency and perceived sensation intensity (PSI) scores during the 10 min after the injection of glutamate (G) and isotonic saline (IS) into the temporalis muscle. *P < 0.05, #*P* < 0.001, ANOVA and McNemar’s test.

22/30 participants (73%) experienced referred sensations during the glutamate injection, in contrast to 10/30 participants (33%) during the isotonic saline injection (Fig. 3). Referred sensation frequency during the glutamate injection was significantly higher when compared to the isotonic saline injection (McNemar, *P* < 0.05) (Fig. 3).

### Perceived sensation intensity following palpation of the masseter muscle

**Figure 4:**
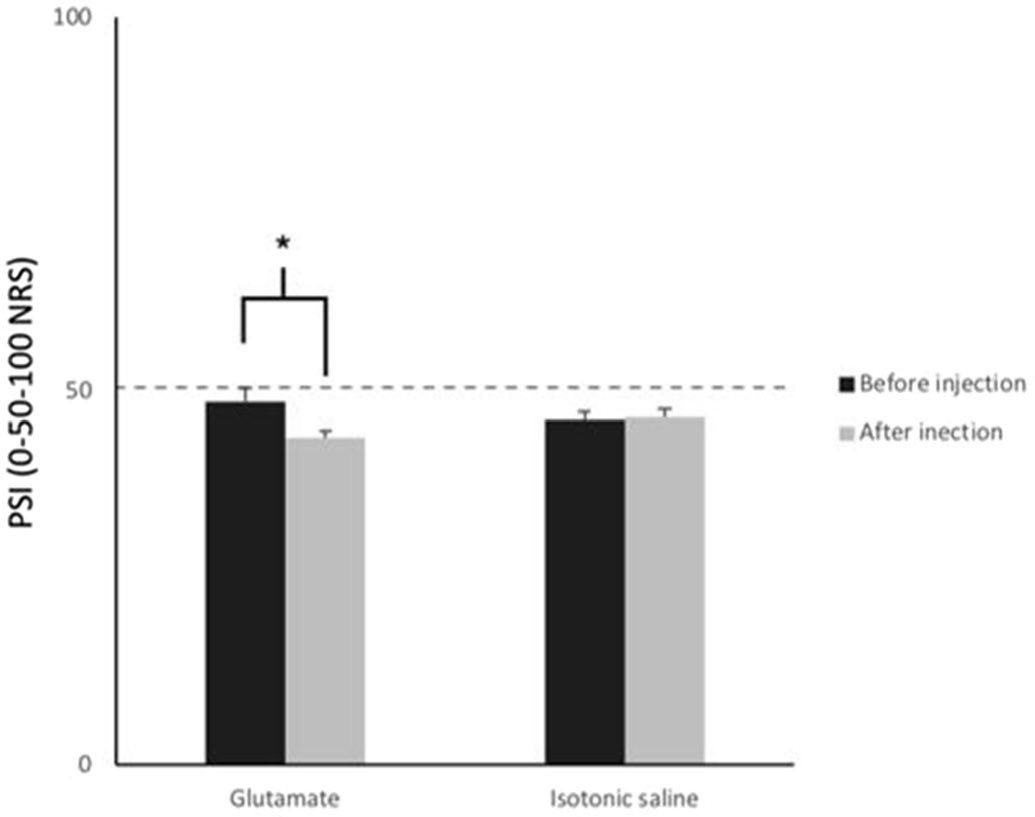
Perceived sensation intensity following mechanical stimulation of the masseter muscle. The vertical bars represent the mean perceived sensation intensity (PSI) scores recorded during the palpation of the masseter muscle. A statistically significant difference was found between the before glutamate and after glutamate session, *P < 0.05, ANOVA. Error bars represent the standard error of the mean.

Regarding the mechanical sensitivity assessment of the masseter muscle, a statistically significant difference was found for the interaction substance × session (3-way ANOVA, substance × session, F (1, 29) = 4.83, *P* = 0.03). Post hoc tests showed that a significant decrease in perceived sensation intensity scores of the masseter muscle occurred after the glutamate injection but not after isotonic saline injection (Tukey HSD *P* = 0.02) (Fig. 4). Regarding the injections, statistically significant higher perceived sensation intensity scores of the temporalis muscle were found for glutamate when compared to the isotonic saline injection (2-way ANOVA, substance × time, F (9, 261) = 13.32, *P* < 0.001) and this difference was present for all time-points (Tukey HSD *P* = 0.02, *P* < 0.001) (Fig. 3).

### Perceived sensation intensity and referred sensation intensity of the temporalis muscle

A moderate positive correlation was found between perceived sensation intensity in the temporalis muscle and referred sensation intensity following injection into the temporalis muscle after the glutamate injection (Pearson, *r* = 0.407, *P* < 0.05). In contrast, no correlation was observed between the perceived sensation intensity and referred sensation intensity scores recorded in the 10 min after the IS injection (Pearson, r = − 0.128, *P* = 0.397).

## Discussion

The main findings of the current study were: (1) referred sensation frequency, location and area following palpation of the masseter muscle were not altered by a painful stimulus to the temporalis muscle; (2) a significant decrease in mechanical sensitivity of the masseter muscle was seen after glutamate injection into the temporalis muscle but not after isotonic saline injection (3) a significant positive relation was seen between perceived sensation intensity scores and referred sensation frequency and intensity scores for the temporalis muscle following glutamate injection.

### Referred sensations

In comparison to previous research on healthy participants^9,23^ there was overall a very high percentage of participants experiencing referred sensations during mechanical stimulation and chemical stimulation. However, the baseline percentages of participants experiencing referred sensations during mechanical stimulation were comparable to the data of Masuda et al.^9^. The high percentage of referred sensation prevalence might be attributed to the fact that participants underwent multiple rounds of mechanical stimulation which led to referred sensations being triggered in different participants every round, thus resulting in a high overall referred sensation prevalence.

In accordance with previous studies, a higher number of participants reported referred sensations when using the more intense forces (2 kg and 4 kg) compared to less intense forces (0.5 kg and 1 kg)^9,13^. Finally, there were no differences in referred sensations before and after injections for either substance. This indicates that painful stimulation of a remote site did not alter the frequency of referred sensations, i.e., it was not possible to prime the occurrence of a referred sensation through a heterotopic painful stimulation.

In addition, there was no difference in the location of referred sensations or area of referred sensations before and after injections and between the two injected substances. This meant that a painful stimulus was not able to alter referred sensation location or increase the referred sensation area. A previous study has shown that the frequency and area of referred sensations during mechanical stimulation was increased following hypertonic saline injection into the infraspinatus muscle when compared to before the injection^11^. Since the duration of the painful stimulus in both the above-mentioned study and the present study were similar, it could be inferred that applying a homotopic painful stimulus before mechanical stimulation causes changes in the central nervous system such as unmasking of latent synapses^26^ in a different manner than a heterotopic painful stimulus. Finally, a previous study has shown that pain refers more frequently to a site of previous pain following pressure-induced pain^16^. These results are in contrast to the present study where referred sensations did not occur more frequently in the temple region following a painful stimulus. The differences seen could be explained by two different factors. The first being that our experimental pain model was not persistent enough to cause changes in the central nervous system that would lead to facilitation of referred sensations to the temple. The second being that these differences could be explained by differences between the trigeminal and spinal system. Future studies assessing referred sensations should take this into consideration.

Finally, there was a significantly higher referred sensation frequency for the glutamate injections into the temporalis muscle when compared to the isotonic saline injections. Since this has not been assessed previously, we cannot compare our results with previous studies. However, these results are in accordance to a previous study that showed the same results for the masseter muscle^13^.

### Perceived sensation intensity

Also in accordance with the previous studies^22,23^, we found a statistically significant difference in perceived sensation intensity between the different forces applied during mechanical pressure stimulation, with higher forces eliciting higher scores for perceived sensation intensity. It can also be noted that on average, the palpation with the 2 kg and 4 kg palpometer caused perceived sensation intensity above the pain threshold, but palpations with the 0.5 kg and 1 kg palpometer did not. In other studies, the average perceived sensation intensity scores after palpation with 2 kg were also above the pain threshold^22,23^.

Furthermore, we found that the perceived sensation intensity scores for the masseter muscle when glutamate was injected in the temporalis were higher in the baseline session than the post-injection session but not when isotonic saline was injected. This finding could be attributed to endogenous pain modulation that occurred following the glutamate injection to the temporalis muscle. The endogenous opioid system has been shown to alter pain perception after prolonged painful stimuli, and this may have occurred following glutamate injection in temporalis muscle^27^.

In the current study, it seems that glutamate-evoked pain in the temporalis lasted on average 6 min and thus longer than what has been shown for the masseter muscle in a previous study^13^. Previous research has found differences in glutamate-induced pain between the masseter and temporalis for other parameters such as peak pain, pain duration and AUC with all of them being increased for the temporalis muscle^21^. These findings could be due to differences in expression of N-methyl-D-aspartate (NMDA) receptors between the masseter and temporalis muscles or related to biophysical characteristics of the muscles.

### Perceived sensation intensity and referred sensation intensity

In the current study, we found a moderate positive correlation between the perceived sensation intensity and the referred sensation intensity following glutamate injection into the temporalis muscle. This result is in slight contrast to a previous study where a low correlation was found between these two parameters when injecting glutamate into the masseter muscle^13^. However, this finding seems to be in accordance with previous data where a clear correlation between the perceived sensation intensity and referred sensation intensity in the region of the anterior tibialis muscle was found^1^. As such, it can be speculated that the relationship between perceived sensation intensity and referred sensation intensity varies from muscle to muscle both within the trigeminal system but also between the trigeminal and spinal systems.

## Limitations

Blinding of the participants and examiners proved to be difficult due to the painful stimuli that were applied. In some cases, the participant knew what substance was injected due to the painful nature of the glutamate injection. Vice versa, examiners knew what substance was injected based on the reaction of the participant. Nevertheless, we believe that this is not a significant limitation in the interpretation of the present findings.

The anatomical map of the head has been used in the DC/TMD to assess locations of the pain^18^. It has proven to be a reliable way for patients and participants to indicate locations of pain^28^. However, the anatomical map of the head does not take the depth of the location of the sensations into account. Future research might benefit from a different 3D-map, giving a more accurate picture of the referred sensations.

## Clinical relevance

This study contributes to the research conducted to investigate the mechanisms behind referred sensations and will hopefully help patients suffering from TMD pain and other musculoskeletal pain conditions that experience referred sensations. From a clinical perspective, this study indicates that short-term pain such as a tooth-ache is not likely to alter the characteristics of referred sensations originating from the masseter muscle. In addition, that the masseter and temporalis muscles react differently to painful stimuli which should be taken into consideration when evaluating myofascial orofacial pain patients, particularly during palpation of the muscles.

For future studies, we recommend using a substance that causes longer lasting sensitization such as NGF, and to conduct a study with a similar experimental protocol to ascertain if with longer sensitization referred sensation can be modified in terms of both frequency but also location. Importantly, the present study confirms the suggestion that referred pain and sensations are epiphenomena of deep tissue pain, i.e., they may not be dependent on pathophysiological processes such a peripheral or central sensitization but rather reflect normal physiology of deep nociceptive processing^11,13,23,24^.

## Conclusion

In the current study, location, area and frequency of referred sensations following mechanical stimulation of the masseter muscle were not altered by the application of a painful stimulus to the temporalis muscle. In addition, there seems to be a positive relationship between painful stimuli and referred sensations frequency and intensity elicited from the temporalis muscle.

## Data Availability

The datasets generated during and/or analysed during the current study are available from the corresponding author on reasonable request.
